# Protocol: a simple method for extracting next-generation sequencing quality genomic DNA from recalcitrant plant species

**DOI:** 10.1186/1746-4811-10-21

**Published:** 2014-06-27

**Authors:** Adam Healey, Agnelo Furtado, Tal Cooper, Robert J Henry

**Affiliations:** 1 Queensland Alliance for Agriculture and Food Innovation, University of Queensland, Brisbane 4072, Australia

**Keywords:** DNA extraction, Next-generation sequencing, CTAB, *Corymbia*, *Coffea*

## Abstract

Next-generation sequencing technologies rely on high quality DNA that is suitable for library preparation followed by sequencing. Some plant species store large amounts of phenolics and polysaccharides within their leaf tissue making genomic DNA extraction difficult. While many DNA extraction methods exist that contend with the presence of phenolics and polysaccharides, these methods rely on long incubations, multiple precipitations or commercially available kits to produce high molecular weight and contaminant-free DNA. In this protocol, we describe simple modifications to the established CTAB- based extraction method that allows for reliable isolation of high molecular weight genomic DNA from difficult to isolate plant species *Corymbia* (a eucalypt) and *Coffea* (coffee). The simplified protocol does not require multiple clean up steps or commercial based kits, and the isolated DNA passed stringent quality control standards for whole genome sequencing on Illumina HiSeq and TruSeq sequencing platforms.

## Introduction

With the advent of next-generation sequencing (NGS) technologies, investigation into the genomes of important industrial plant species has never been easier or more economical. Traditionally, genome analysis has relied on relatively small amounts of DNA of moderate purity for the purpose of restriction enzyme fingerprinting or polymerase chain reaction (PCR) amplification. However, NGS technologies, such as the Illumina HiSeq and TruSeq platforms, allow for the investigation of the entire genome of plants and as such require the input of several micrograms of high quality DNA. In the context of NGS, high quality DNA is characterized as DNA that is predominantly high molecular weight with an A260/280 ratio between 1.8 and 2.0 and without contaminating substances, such as polysaccharides or phenolics [1,2], which impede or inhibit DNA library preparation for NGS. The quality and quantity requirements for plant DNA sequencing by NGS often confine extraction methods to using leaf material, which is problematic due to the accumulation of high amounts of phenolics and polysaccharides within a variety of species [3,4]. Polysaccharides, due to their chemical properties, co-precipitate with genomic DNA, giving solutions a viscous, glue-like appearance [5] and are known to inhibit the NGS library preparation (Nock C, personal communication). Phenolics, such as terpenoids and tannins, undergo rapid oxidation upon their release from leaf tissue and irreversibly bind to the phosphate backbone of DNA, characterized by the browning of leaf material [6,7]. Both contaminants prevent the use of DNA for molecular biology purposes, such as PCR, restriction digests, or sequencing by inhibiting the action of polymerases or endonucleases [8,9]. Forest trees, such as *Corymbia*, and species belonging to the *Coffea* genus also accumulate these contaminants in their leaves, limiting the study of their genomes [10-12].

The majority of DNA extraction methods from plant leaf tissue are derived from the original hexadecyltrimethylammonium bromide (CTAB) based method, described by Doyle and Doyle in 1987 [13]. To contend with the problems associated with phenolics and polysaccharides, the protocol has been modified to include polyvinylpyrrolidone (PVP) and high salt solutions to isolate genomic DNA [5,6,8,9,14,15]. Unfortunately, these methods can be time consuming, either relying on long incubation steps, nuclei pre-extraction that increases handling time, or requiring multiple DNA washes and precipitations that decrease overall yield. For NGS library preparation, DNA from difficult-to-isolate plant species often require commercial kit based methods to supplement CTAB based extractions to generate genomic DNA of high enough quality to pass stringent conditions for library preparation [4,16]. Kit based extraction methods are intended to easily remove contaminants, but are often expensive, particularly when many samples are required for analysis. The problem of losing DNA through subsequent column washes or precipitations can be exacerbated when only small amount of leaf tissue is available for collection.

NGS quality control requirements are often very stringent and require preparation of DNA that is of high molecular weight with little evidence of band shearing, containing no evidence of contamination from protein, RNA or polysaccharides, and has a 260/280 nm absorbance ratio of approximately 1.8-2.0. A fast, simple, and reliable DNA extraction method that does not rely on long incubations, multiple DNA precipitations, or supplementation of commercial supplies or reagents to meet next-generation library preparation requirements will be invaluable to plant research. The method described below illustrates how the addition of PVP alone to an established CTAB based method does not necessarily translate to an effective DNA extraction protocol, and demonstrates how subtle manipulations to an extraction protocol can isolate high quality genomic DNA from recalcitrant plant species, free of contamination and suitable for NGS library preparation.

## Materials and methods

### Consumables

50 mL Falcon Tubes

RNAse A (Sigma Cat No. R6513)

Polyvinylpyrrolidone (PVP) (Sigma Cat No. PVP10) (not required)

Chloroform: Isoamyl alcohol 24:1 (Sigma Cat No. C0549)

Liquid nitrogen

β-mercaptoethanol (Sigma Cat No. 63689)

Trizma base (Sigma Cat No. 1503)

Ethylenediaminetetraacetic acid disodium salt dihydrate (EDTA) (Chem Supply Cat No. EA023)

Agarose (Amresco Cat No. 0710)

Sodium Chloride (NaCl) (Ajax Finechem Cat No. 1103414)

Hexadecyltrimethylammonium bromide (CTAB) (Sigma Cat No. 52365)

*Eco*RI (not required- used for quality assurance) (NEB Cat No. R0101S)

*Hin*dIII-HF (not required- used for quality assurance) (NEB Cat No. R3104S)

### Reagents

Extraction Buffer: 100 mM Tris-HCl (pH 7.5), 25 mM EDTA, 1.5 M NaCl, 2% (w/v) CTAB, and 0.3% (v/v) β-mercaptoethanol- added immediately before use

RNAse A stock solution (10 mg/mL)

5 M NaCl

95% ethanol (v/v)

70% ethanol (v/v)

TE Buffer: 10 mM Tris-HCl (pH 7.6), 0.1 mM EDTA

CutSmart Buffer (NEB Cat No. B7204S)

NEBuffer EcoRI (NEB Cat No. B0101S)

Equipment

Mortar and Pestle

Water Baths (65°C and 37°C)

Centrifuge (capable of spinning 50 mL centrifuge tubes at 5000 × g)

-20°C Freezer

Gel electrophoresis system (e.g. Jordan Scientific JP-250)

NanoDrop UV/Vis Spectrophotometer (e.g. NanoDrop 8000, Thermo Scientific)

### Plant material and tissue collection

Leaf tissue of *Corymbia citriodora* subsp. *variegata, Corymbia henryi, Corymbia torelliana, and Corymbia citriodora* subsp. *citriodora* was obtained from Queensland Department of Agriculture, Fisheries and Forestry in Gympie, Australia. Leaf tissue of *Coffea brassii* was obtained from the Australian Tropical Herbarium in Cairns, Australia. Leaf material after harvesting was transported on ice and stored at -80°C until subjected to DNA extraction.

### Protocol

#### Preparatory steps

Before grinding, pre-chill the mortar and pestle (to minimize frozen tissue thawing) and 95% ethanol solution at -20°C. Pre-heat water baths (65°C and 37°C) before beginning the extraction. Once pre-heated, prepare 10 mL (per 1 g of leaf tissue) extraction buffer by adding 0.3% (v/v) β-mercaptoethanol in a 50 mL Falcon tube, and pre-heat in the 65°C water bath. PVP can also be added at this point, but is not required.

#### Grinding and tissue disruption

Using liquid nitrogen, grind 1 g of frozen leaf tissue into a fine powder. Place the powder into a new 50 mL Falcon tube and mix in the pre-heated extraction buffer. Put the sample into the 65°C water bath and mix by inversion every 10 min for 30 min- 1 h. After incubation, centrifuge the sample tube for 5 min at 5000 × g (to pellet and remove un-lysed leaf tissue) and decant the supernatant into a new 50 mL Falcon tube.

#### Protein Extraction and RNAse treatment

Add 1 volume of chloroform: isoamyl alcohol to the solution and mix by inversion for 5 min. Centrifuge the sample for 10 min at 5000 × g and pipette the upper aqueous phase into a new Falcon tube, taking care to avoid the aqueous/organic layer interface. Add 5 μL of RNAse A (10 mg/mL) to the solution and incubate at 37°C for 15 min with periodic, gentle mixing. After incubation, add 1 volume of chloroform: isoamyl alcohol to the solution and mix by inversion for 5 min. Centrifuge the solution for 10 min at 5000 × g and pipette the aqueous phase into a new Falcon tube, again taking care to avoid the organic layer.

#### Precipitation

Add ½ volume of 5 M NaCl to the sample and mix gently by inversion. Then, add 3 volumes of cold 95% ethanol and mix gently by inversion. Place the tubes into a -20°C freezer and incubate for 1 h. NOTE: do not leave the sample at -20°C for more than 1 h as both the CTAB and NaCl can precipitate from solution, preventing DNA isolation.

After incubation, centrifuge the Falcon tube for 10 min at 5000 × g to pellet the DNA. Carefully decant away the supernatant and wash the DNA pellet with 3 mL of 70% ethanol. Gently swirl the solution and centrifuge again for 10 min at 5000 × g. Carefully decant the supernatant and air-dry DNA pellet for 15 min at room temperature. Once dried, suspend DNA in 200 μL of TE buffer.

#### DNA quality and quantity assessment

Assess the quality of the extracted DNA using a NanoDrop UV/Vis spectrophotometer and 0.7% (w/v) agarose gel, looking for a single absorbance peak at 260 nm, a 260/280 absorbance ratio of 1.8-2.0, and no evidence of substantial band shearing or contamination (either RNA or polysaccharide).

### Comments

Since the advent of the CTAB-based extraction method from plant leaves by Doyle and Doyle in 1987, many different iterations have been published, each with modifications to contend with the co-extractives of polyphenolics and polysaccharides present in the leaves of many plant species [3,5-8,15]. While having demonstrated their effectiveness for isolating DNA that is suitable for PCR amplification or restriction digests, all methods currently published in the literature require long incubations, and multiple precipitation steps and ethanol washes to produce RNA-free genomic DNA of high purity. As next-generation sequencing requires large amounts of high quality DNA, each additional precipitation and wash increases handling time and lowers overall yield. Commercial column based extraction kits, such as DNeasy (Qiagen, Australia) or Wizard (Promega, Australia), are effective for isolating contaminant free DNA from recalcitrant plant species, including eucalypts [4,16]. However, commercial kits can be expensive and carry the risk of losing DNA on the column, which in turn necessitates several extractions followed by pooling of DNA.

To test the modifications made to the extraction method (NGS protocol) against the well-established original CTAB method (used routinely in our laboratory to reliably extract high quality DNA from rice, sugarcane, barley and wheat for sequencing [17,18]), six grams of frozen *Corymbia citriodora* subsp. *variegata* leaf tissue was ground and aliquoted evenly into the extractions described below. The quality of DNA from each extraction was verified spectrophotometrically using a NanoDrop instrument and agarose gel electrophoresis. The NanoDrop absorbance profile is useful for detecting contamination such as protein, salts or polysaccharides, all of which can inhibit NGS library preparation. High quality DNA is characterized as having a 260/280 nm absorbance ratio of approximately 1.8, with a single absorbance peak at 260 nm. The spectrophotometric profile is also useful for detecting phenolic oxidation, as the aromatic structure will absorb at 230 and 270 nm [1]. If oxidation is suspected to have occurred, endonuclease digestion can be used to further assess DNA quality before library preparation as phenolics, which inhibit polymerases, also inhibit restriction enzymes [8,9].

Visualization of DNA on an agarose gel provides evidence of band shearing and RNA and polysaccharide contamination. Mechanical disruption, such as vortexing, causes DNA strands to shred apart, indicated by a wide DNA band with poor resolution. NGS library submission requires intact, high molecular weight genomic DNA, so all solution mixing steps were done by gentle inversion. Gel electrophoresis is also beneficial for visualizing RNA and polysaccharides, both of which contaminate sequencing reactions. RNA is evident as a distinct banding pattern at various sizes throughout the gel, whereas polysaccharides will migrate quickly and conglomerate at the bottom of the gel as a non-distinct fluorescent structure. Yield was determined through relative band intensity approximation with 100 and 200 ng λ DNA standards, as the NanoDrop concentration readings can inflate yield of genomic DNA.

### Traditional CTAB extraction method

**Figure 1:**
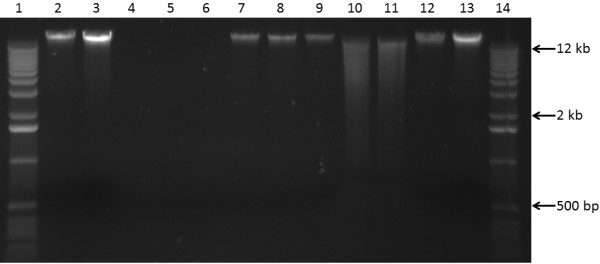
**Genomic DNA preparation of *Corymbia citriodora *subsp. *variegata *resolved by electrophoresis.** 1 kb DNA ladder (1, 14), 100 and 200 ng λ DNA standards (2, 3 and 12, 13) respectively. DNA extractions using the traditional CTAB-based method with no PVP (4), 1% PVP (5), and 4% PVP (6). DNA extractions using the NGS protocol with no PVP (7), 1% PVP (8), and 4% PVP (9). Endonuclease digestions of DNA extracted without PVP with *Eco*RI (10) and High-Fidelity *Hin*dIII (11). Results from six grams of leaf tissue finely ground using a mortar and pestle, then aliquoted (1 g) for each extraction. DNA was resolved by electrophoresis in a 0.7% agarose gel and visualized using SYBR Safe DNA gel stain. Percentages are represented as w/v. CTAB: hexadecyltrimethylammonium bromide; PVP: polyvinylpyrrolidone.

**Figure 2:**
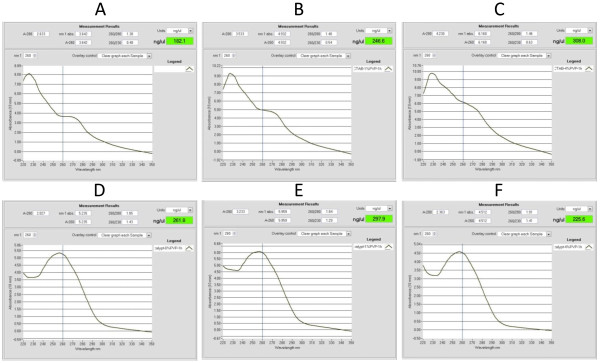
**NanoDrop measurement profile of genomic DNA extractions from *Corymbia citriodora *subsp. *variegata*.** DNA extractions using a traditional CTAB-based method with **(A)** no PVP, **(B)** 1% PVP and **(C)** 4% PVP. DNA extractions using the NGS protocol with **(D)** no PVP, **(E)** 1% PVP, and **(F)** 4% PVP. Results from six grams of leaf tissue finely ground using a mortar and pestle, then aliquoted (1 g) for each extraction. Percentages are represented as w/v. CTAB: hexadecyltrimethylammonium bromide; PVP: polyvinylpyrrolidone.

Using the original CTAB protocol, we were unsuccessful in isolating DNA from *Corymbia* leaves. During incubation at 65°C, the extraction solution began darkening, eventually turning brown. Upon precipitation, despite the observation of a small, brown pellet, the agarose gel failed to reveal any DNA (Figure 1, lane 4). The UV/Vis spectrophotometer absorbance peaks at 220-230 nm and 270-280 nm (Figure 2A) are likely due to polysaccharide, phenol and aromatic co-extractives [1]. The browning of solution has been attributed to the oxidation of phenolic secondary metabolites in plant leaves [4,6,19,20], a known problem with *Corymbia*[11,12] and *Coffea*[10].

The addition of PVP into CTAB based extractions to absorb phenolics, preventing their oxidation that renders DNA unusable for downstream application, has been used successfully for other recalcitrant plant species [4,5,7,9], typically at a concentration of 1-2% (w/v). The addition of 1% and 4% PVP to the traditional CTAB extraction method failed to isolate any useable DNA from *Corymbia citriodora* subsp. *variegata*. Again, the browning of solution occurred, and upon precipitation, a minute brown pellet was observed for each extraction. NanoDrop measurements revealed the persistence of the contamination absorbance peaks of 220-230 nm and 270-280 nm (Figure 2B-C). When resolved on a 0.7% agarose gel, no DNA was observed (Figure 1, lanes 5-6).

### NGS extraction protocol

The NGS protocol allowed for the isolation of high quality DNA from *Corymbia citriodora* subsp*. variegata*. The NanoDrop spectrophotometer measurement profile showed a single absorbance peak at 260 nm, and a 260/280 ratio of 1.85 (Figure 2D) Gel electrophoresis revealed a single, high molecular weight DNA band with little evidence of shearing and no RNA or polysaccharide contamination (Figure 1 lane 7). To further evaluate the quality of the extracted genomic DNA, approximately 1 μg (per reaction) was digested overnight at 37°C with restriction enzymes *Eco*RI and High-Fidelity *Hin*dIII (New England BioLabs, Ipswich Massachusetts). Resolution of the digests on the agarose gel revealed efficient endonuclease activity of both enzymes (Figure 1 lanes 10 and 11, respectively).

The spectrophotometric profile and yield varied little when increasing amounts of PVP (1% and 4% w/v) were added to the extraction buffer. Each DNA extraction had a 260/280 absorbance ratio of 1.84 and 1.91 respectively (Figure 2E-F), and high molecular weight DNA band with little shearing or contaminants (Figure 1 lanes 8-9). Based on relative band intensity of the 2 μL of sample resolved on the gel with the 100 ng λ DNA standard, the method consistently yielded approximately 5 μg of DNA per gram of leaf tissue. Although the A260/230 ratios (a secondary measure of DNA quality) [1] for the extractions were lower than expected (1.41, 1.29, and 1.43 respectively), these results in combination with the endonuclease digestions suggested that PVP was not required to prevent phenolic oxidation, and the protocol was suitable for the isolation of DNA for whole genome NGS library preparation and sequencing. The modifications and considerations for the protocol are discussed below.

### Modifications

The modifications to the previously cited methods were designed to simplify the protocol and maximize DNA yield by reducing the number of handling steps, DNA precipitations, and washes required, and eliminating the need for long incubations or supplementation with commercial based kits and reagents.

### Phenolic oxidation

As shown (Figure 1 lanes 7-11 and Figure 2D-F), PVP was not required to prevent phenolic oxidation which renders DNA unsuitable for use. This is likely due to the presence of β-mercaptoethanol, a reducing agent [2], and the centrifugation step after 65°C incubation. The centrifugation and pelleting of un-lysed leaf material for removal was included to reduce the continued leeching of leaf phenolics into solution. Also, as un-lysed leaf tissue settles at the interface between the aqueous and organic phases during the first protein extraction step, its early removal increases the clarity between the two phases allowing easier pipetting of the aqueous portion. After centrifugation, chloroform: isoamyl alcohol was added as quickly as possible for further separate phenolics from the aqueous portion. If PVP has been added for phenolic absorption, the first protein extraction step will remove the majority, while the remainder is removed by the second extraction.

### RNAse treatment

RNAse A treatment, a requirement for the isolation of high quality genomic DNA, is traditionally added after the DNA has been precipitated, washed and dissolved into a stabilizing buffer which necessitates additional steps to remove the enzyme and re-precipitate and wash the DNA. Each additional handling step and precipitation may produce DNA of higher quality, but decreases overall yield as typically, the simplest method for extraction will provide the most reliable result [21]. In this protocol, RNAse A was added between the two chloroform: isoamyl alcohol solvent extractions to allow for a single DNA precipitation step at the end of the protocol. As two washes using chloroform: isoamyl alcohol are required for high quality DNA extraction, the addition of the RNAse and the 15 minute incubation at 37°C after the first solvent extraction efficiently digests RNA, while the second solvent extraction removes the enzyme. This eliminates the need for further treatments, precipitations and washes once the DNA was re-suspended in TE buffer during the final step of the procedure.

### Precipitation

Included with the protocol was the addition of a high salt solution before DNA was precipitated with 95% cold ethanol. Polysaccharides have a similar solubility to DNA and co-precipitate in either isopropanol or ethanol, inhibiting downstream molecular application [4]. The addition of a high salt buffer increases their solubility in ethanol, allowing their removal once the DNA has been precipitated and pelleted [22]. During the -20°C precipitation step, the 1 h incubation time should not be exceeded as NaCl and CTAB will eventually precipitate, preventing the DNA pellet from forming during centrifugation.

### NGS library submission, preparation and sequencing

Based on the success of the method, the NGS protocol was applied to other samples of intended for NGS library preparation and sequencing. Due to limited amounts of leaf tissue available, 4% PVP was included with the extraction for full confidence that no phenolic oxidation would occur. High quality DNA was extracted from *Corymbia citriodora* subsp. *citriodora*, *Corymbia henryi*, *Corymbia citriodora* subsp*. variegata* and *Corymbia torelliana* for NGS library preparation and sequencing. The *Corymbia* genomic DNA samples were submitted to the Joint Genome Institute (JGI) for library preparation and sequencing on the Illumina HiSeq 2500 platform and passed their quality control measures which require: high molecular weight genomic DNA free of polysaccharide, RNA and protein contamination, and a 260/280 nm absorbance ratio between 1.6 and 2.2.

**Figure 3:**
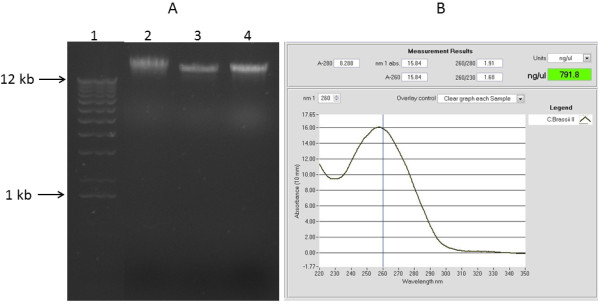
**DNA quality and yield assessment for *Coffea brassii *genomic DNA using the NGS extraction protocol. A)** Genomic DNA preparation of *Coffea brassii* resolved by electrophoresis. 1 kb DNA ladder (1), 100 ng and 200 ng λ DNA standards respectively (3, 4), and DNA extraction using the modified NGS extraction protocol (2). DNA was separated by electrophoresis in a 0.7% agarose gel and visualized using SYBR Safe DNA gel stain. The gel image was cropped to exclude an unrelated sample. **B)** NanoDrop measurement profile of *Coffea brassii* leaf extracted genomic DNA using the modified NGS extraction protocol.

The robustness of the NGS protocol was demonstrated with another recalcitrant plant genus, *Coffea*[10], to isolate high quality DNA from *Coffea brassii* for sequencing. As only 0.1 grams of *C. brassii* leaf material was available, the protocol was modified to use 5 mL of extraction buffer and 40 μL of TE buffer to resuspend the DNA pellet. The DNA extraction was successful, the NanoDrop spectrophotometer profile showing a single 260 nm absorbance peak and a 260/280 nm absorbance ratio of 1.91 (Figure 3B). Resolution of 2 μL of DNA by gel electrophoresis revealed high molecular weight DNA with little evidence of shearing and no observable contamination (Figure 3A). Based on relative band intensity with the 100 ng λ DNA standard, approximately 1.5-2 μg of DNA was isolated, indicating a theoretical yield of 15-20 μg of DNA per gram of leaf tissue. The *C. brassii* DNA sample was submitted to the Australian Genome Research Facility (AGRF) for library preparation and paired end sequencing on the Illumina TruSeq platform.

A summary of the DNA extractions for NGS library preparation and sequencing by JGI and AGRF is provided in Table 1.

**Table 1:** **Summary of *Corymbia *** **and *Coffea *** **genomic DNA extractions and sequencing results from JGI and AGRF**

**Species**	**Absorbance 260/280 nm**	**Quantity of DNA submitted (μg)**	**NGS quality control results**	**Number of sequencing reads**
*Corymbia henryi*	1.78	19.1	Pass (JGI)	206,959,160 (HiSeq)
*Corymbia citriodora* subsp. *citriodora*	1.81	4.4	Pass (JGI)	169,513,988 (HiSeq)
*Corymbia citriodora* subsp. *variegata*	1.82	5.5	Pass (JGI)	234,021,522 (HiSeq)
*Corymbia torelliana*	1.93	6.3	Pass (JGI)	213,411,194 (HiSeq)
*Coffea brassii*	1.91	1.5	Pass (AGRF)	145,197,482 (TruSeq)

### Sequencing quality

As sequencing is influenced by the quality of DNA provided, raw Illumina reads from JGI and AGRF were assessed using read quality distributions, generated by CLC Bio Genomics WorkBench, software version 5.5.2 (CLC Bio, Denmark). Read quality distributions are based upon PHRED quality scores, which estimate the probability of error per base call [23]. The read quality distribution visualizes this data for the entire sequencing library, normalized to the total number or sequences.

**Figure 4:**
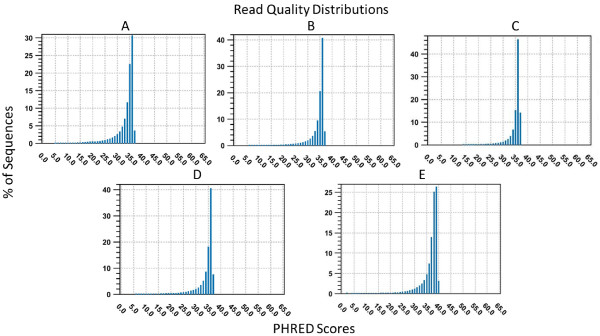
**Paired end read quality distributions of *Corymbia *and *Coffea *samples from Illumina HiSeq and TruSeq library preparations. (A) ***Corymbia citriodora* subsp. *citriodora*, **(B) ***Corymbia henryi*, **(C) ***Corymbia torelliana*, **(D) ***Corymbia citriodora* subsp. *variegata*, and **(E) ***Coffea brassii*. The x-axis represents the PHRED quality score and the y-axis represents the percentage of sequences with a particular score, normalized to the total number of sequences. The distribution graph was generated using CLC Bio software (Version 5.5.2).

Each *Corymbia* sequencing library (prepared by JGI, filtered above a PHRED score of 5) and the *C. brassii* sequencing library (prepared by AGRF) produced over 100 million reads per library, with a modal PHRED quality score of 36 and 39, respectively (Figure 4A-E). This represents a base call accuracy of approximately 99.999%, providing high confidence in the quality of DNA submitted. Despite lower A260/230 ratios for the submitted *Corymbia* (~1.4) and *Coffea* (1.68) samples, there were no observable differences between the library preparation and sequencing quality for the two species on either sequencing platform.

## Conclusion

The described method, developed to improve genomic DNA extractions from leaf tissue of recalcitrant plant species, is a marked improvement over other methods as it does not require multiple clean up steps, precipitations, or commercial based kits or reagents. Using the protocol, high quality DNA was isolated from species of *Corymbia* and *Coffea* that passed stringent Illumina NGS library submission requirements, despite high amounts of high leaf phenolics and polysaccharides. The method was generated with the intent of using a single protocol for all plant species, regardless of the presence or absence of DNA co-extractive contaminants. With this robust protocol, whole-genome sequencing is possible from recalcitrant plant species using established DNA sequencing technologies for advanced bioinformatics investigations.

## Competing interests

The authors declare they have no competing interests.

## Authors’ contributions

AH isolated genomic DNA from *Corymbia* and drafted the manuscript with sections written by AF. AH, AF, and RH developed and optimized the NGS protocol. TC extracted genomic DNA from *C. brassii* using the NGS protocol. All authors read and approved the final manuscript.
